# News and Notes

**Published:** 1906-07

**Authors:** 


### *NEWS AND NOTES.*

---

Dr. J. H. Knight died May 19th, at his home in Eatonton, Ga.

---

The Philippine Medical School is soon to be opened at Manila.

---

The art of pulse-taking in modern times dates only from 1707.

---

Dr. Thos. E. Fortson has located at Brood, Wilkes county, Georgia.

---

One thousand physicians were rendered helpless by the great San Francisco fire.

---

Jefferson Medical College celebrated its eighty-first annual commencement June 4.

---

Augusta Orphan Asylum has decided to sell its present property and purchase a new site.

---

The University of Leipsic is arranging a celebration for 1909 of its five-hundredth anniversary.

---

Edward S. Harkness has given $2,700 to the morphologic museum at the Columbia Medical School.

---

Servian student statistics show that men in that country are dropping medicine, while women are turning to this profession for a livelihood.

Dr. Herbert Fox has been elected director of the pathological laboratory of the Pennsylvania Zoological Society.

---

According to a recent monthly report of the Board of Health of the Philippine Islands, the number of lepers now living in the archipelago is 3,683.

---

Battle & Co. has just issued the tenth of the series of twelve illustrations, of the Intestinal Parasites, and will send them free to physicians on application.

---

The University of Berlin has inaugurated a course of study on alcoholism which is to comprehend every phase of the subject, moral, legal, social, hygienic, pathologic, etc.

---

Dr. Jokichi Takamine has been decorated by the Mikado with the Fifth order of the Rising Sun in consideration of his researches and discoveries in applied chemistry.

---

The office of publication of the *Journal of the South Carolina Medical Association* has been changed from Charleston to Greenville. Dr. J. Wilkinson Jervey is now editor of the *Journal.*

---

A controversy between the New York State Charities Board and the trustees of the Bellvue and the Allied Hospitals concerning the plans for the new hospital will tie up the work for some time.

---

At the annual meeting of the Alumni Association of the University of Maryland School of Medicine, June 1, Dr. Randolph Winslow, Baltimore, delivered the oration on "The University in 1871 and 1906."

THE Walter Reed United States Army General Hospital, to be erected in the District of Columbia, is to be named in honor of the great physician who demonstrated the transmission of yellow fever by mosquitoes.

---

A PRELIMINARY examination of applicants for appointment in the medical corps of the army will be held at the various military posts throughout the United States on July 31, 1906. Full information may be obtained from the Surgeon-General of the army.

---

AT the recent meeting of the German Association for Prevention of Venereal Diseases, in Berlin, Dr. Blaschko, the general secretary, reported that the Association had 5,000 members, and that 1,500,000 copies of pamphlets or leaflets published by it had been distributed to the public.

---

ARRANGEMENTS are being made for holding an important Dental Convention at the Jamestown Exposition, Norfolk, Virginia, next year. Not only will the various State Dental Conventions be held here, but some of the European countries will be represented at this National Dental Congress.

---

A NEW tuberculosis serum has been brought forward by Dr. Ishigami, an eminent Japanese bacteriologist. Unlike other antitoxins, this does not induce a rise in temperature. Dr. Ishigami declares that it has cured one-third of the patients experimented upon; it is not, however, applicable to advanced cases.

---

THE Chair of Physiology of Harvard Medical School will be vacant at the end of the present academic year when the resignation of Dr. Henry P. Bowditch, George Higginson, professor of physiology, will take effect. Dr. Bowditch has been connected with the faculty of the school for more than thirty years. For ten years (1883-1893) he was dean of the faculty.

---

DR. RALPH COMER, of Tignall, Wilkes county, Ga., has sold his practice at Tignall to Dr. F. A. Blackwell, of Elberton, Ga. Dr. Comer, after taking a postgraduate course in St. Louis, Mo., will move to Athens, Ga., about September 1, 1906, to do office and general practice. He will devote most of his postgraduate course to electro-therapeutic and other office work.

---

THE seventy-second annual commencement exercises of Tulane University of Louisiana, Medical Department, New Orleans, were held May 3, when a class of 102 received degrees in medicine. The annual report was given by the dean, Dr. Stanford E. Chaille, and the annual address was delivered by Surgeon Joseph H. White, United States Public Health and Marine-Hospital Service.

---

AT the fifty-third annual meeting of the Medical Society of the State of North Carolina, held in Charlotte, May 29, 30 and 31, the following officers were elected: President, Stephen D. Booth, Oxford; vice-presidents, Drs. Charles M. Strong, Charlotte; John E. McLaughlin, Statesville, and William F. Hargrove, Kinston; Secretary, Dr. David A. Stanton, High Point, and treasurer, H. McKee Tucker, Raleigh. Morehead City was chosen as the place of the next annual meeting.

---

THE United States Civil Service Commission announces an examination on July 5-6, 1906, to secure eligibles from which to make certification to fill vacancies in the position of hospital interne (male) under the Isthmian Canal Commission, on the Isthmus of Panama, as they may occur. As an insufficient number of eligibles to meet the needs of the service resulted from the examination held on December 6-7, 1905, for this position, qualified persons are urged to enter this examination. Age limit, 20 to 30 years on the date of the examination; salary, $100 per month, with quarters, but without board and washing.

